# Thyroid testing in primary hypothyroidism

**DOI:** 10.4102/phcfm.v17i1.5043

**Published:** 2025-06-30

**Authors:** Anshula Ambasta, Guillaume Grenet, Jessica Otte, Wade Thompson, Kenneth Bassett, Thomas Perry

**Affiliations:** 1 Department of Anaesthesiology, Pharmacology and Therapeutics, Faculty of Medicine, University of British Columbia, Vancouver, Canada

**Keywords:** diagnostic tests, hypothyroidism, mass screening, overdiagnosis, thyroid function tests, pregnancy, thyroid diseases

## Abstract

Thyroid hormones regulate essential metabolic processes and development. The hypothalamic–pituitary–thyroid axis regulates hormone production, with thyroid-stimulating hormone (TSH) levels being a key indicator of thyroid function in primary hypothyroidism. This therapeutics letter emphasises a TSH-centred approach to the diagnosis and management of primary hypothyroidism (dysfunction at the level of the thyroid gland) in adults. It discourages routine thyroid function screening in asymptomatic individuals due to a lack of demonstrated benefit and potential harm from overdiagnosis and overtreatment. It outlines appropriate diagnostic strategies, including when to use TSH, free T4 (thyroxine), and free T3 (triiodothyronine) tests, and outlines indications for antibody testing. Special considerations are provided for subclinical hypothyroidism and hypothyroidism during pregnancy.

## Vignette

*A 40-year-old woman presents to your clinic with symptoms of weight gain, constipation, fatigue, cold intolerance and brittle nails. Physical exam shows dry skin with excoriations and thin hair. She is concerned about thyroid disease and based on her internet research is requesting you to check her ‘complete thyroid panel’. What test(s) would you order for her?*

## Thyroid hormones

Thyroid hormones are necessary for the development of human tissues and metabolic regulation of nearly all cells.^1^ Hormone production is regulated by a feedback loop along the hypothalamus–pituitary–thyroid axis. The hypothalamus produces thyrotropin-releasing hormone (TRH) which controls production of thyroid-stimulating hormone (TSH) by the anterior pituitary gland. Thyroid-stimulating hormone regulates production and secretion of the two forms of thyroid hormone, thyroxine (T4) and the more bioactive triiodothyronine (T3) by the thyroid gland.^1^ The thyroid gland secretes mainly T4, whereas other tissues convert T4 to T3 at the cellular level.^2^ Intracellular T3 levels regulate pituitary secretion and blood levels of TSH.^3^ Most circulating T4 and T3 is bound to transport proteins in plasma; it is the small free fraction (soluble hormone) that is measured through diagnostic tests.^4,5^

Thyroid-stimulating hormone, free T4 and free T3, ordered in a stepwise fashion, are used to diagnose and monitor disorders of thyroid function: clinical conditions of thyroid hormone deficiency (hypothyroidism) or excess (hyperthyroidism).^1,5^ Tests assess abnormal function of the thyroid gland itself (primary dysfunction) or of its regulation by the pituitary gland or hypothalamus (central dysfunction).^1,6^

Antibody tests can help investigate immune-mediated thyroid dysfunction. Thyroid peroxidase (TPO) antibodies can help confirm a diagnosis of Hashimoto’s thyroiditis as a cause of primary hypothyroidism.^3^ However, this test seldom changes clinical management. There are some clinical situations where measurement of anti-TPO antibody level may be useful.^5^ For example, anti-TPO antibodies may indicate the presence of autoimmune thyroiditis in patients with a goitre or mildly elevated TSH. Being positive for anti-TPO antibodies increases the risk of developing overt hypothyroidism in people with subclinical hypothyroidism or those at risk of developing thyroid dysfunction such as people with autoimmune diseases (e.g. type 1 diabetes), chromosomal disorders (e.g. Turner syndrome or Down syndrome) and people on certain medications such as lithium or amiodarone.^3,5,7^ If performed, and a person is anti-TPO antibody positive, there is no reason to repeat anti-TPO antibody testing.^5^

## Use a thyroid-stimulating hormone-centred approach to diagnose and monitor primary hypothyroidism

### Diagnosis of primary hypothyroidism

Because TSH is regulated by thyroid hormones, TSH levels reliably predict thyroid hormone function.^8^ Unless there is clinical evidence to suspect central thyroid dysfunction (at the hypothalamus or pituitary level), which is rare, TSH should be the principal test to assess for primary thyroid gland dysfunction.^9^ If ‘suspected hypothyroidism or hyperthyroidism’ is selected on the standard out-patient requisition, some clinical laboratories first measure plasma TSH; they proceed to measure free T4 only if TSH is abnormal.^5^ Usually, the free T4 level confirms a diagnosis of hypothyroidism or hyperthyroidism. Free T3 is ordered only in clinically suspected hyperthyroidism when the TSH is suppressed, but the free T4 is not elevated.^5^ The TSH alone is not reliable in the diagnosis of central causes of hypothyroidism (e.g. hypopituitarism).^6,10^

### Monitoring primary hypothyroidism

For a patient with established primary hypothyroidism on levothyroxine monotherapy, TSH is sufficient to monitor replacement therapy. As TSH values change slowly, it is better to wait for at least 6 weeks following a change in thyroid hormone replacement dose before re-checking TSH and dose.^3,11^ Once TSH normalises under treatment, annual repeat TSH suffices, unless there is a clinical change that might impact levothyroxine pharmacokinetics (e.g. pregnancy, significant weight changes, gastrointestinal disorders, food supplements containing calcium and iron).^12^

Serum TSH can vary by up to 40% between measurements – even when sampled at the same time of day – yet not indicate any change in thyroid function.^13^ Thyroid-stimulating hormone also varies by time of day.^14^ Thus, one should expect changes within the reference range that reflect inherent biological variability, but are not clinically meaningful. Thyroid-stimulating hormone also tends to rise with age, although most laboratories do not report age-specific reference ranges. Among 16 533 participants in the 1988–1994 US National Health and Nutrition Survey III, there was a shift towards higher serum TSH in older people. For example, the 97.5th percentile for TSH in people aged 20 to 29 was 3.56 mU/L but rose to 7.49 mU/L for people over age 80. In this older group, 70% of people with TSH > 4.5mU/L were within the reference range for their age.^15^

### Do not screen routinely with thyroid function tests

A 2019 systematic review aiming to evaluate benefits and harms of screening to detect thyroid dysfunction found no studies on screening for thyroid disease.^16^ Screening in asymptomatic adults can lead to unnecessary blood draws, further tests, and treatments that do not confer clinical benefit.^17^ As such, some guidelines recommend against ‘routine’ thyroid function testing in adults.^17^ The Practical Approach to Care Kit (PACK) guideline for adults recommends testing for patients with symptoms suggestive of thyroid dysfunction such as: weakness, tiredness, weight gain, low mood, dry skin or cold intolerance.^18^ Strong recommendations from the Society for Endocrinology, Metabolism and Diabetes of South Africa (SEMDSA) and Association of Clinical Endocrinologists of South Africa (ACE-SA) guideline for the management of hypothyroidism in adults, encourage a high index of suspicion and at the same time recognise that many of the clinical features of hypothyroidism are nonspecific, especially in the elderly.^19^ In addition, guidelines suggest consideration of testing for patients with symptoms and signs that suggest thyroid disease or for people with specific risk factors which include advanced age (men over 60 or women over 50 years), personal or family history of thyroid disease, coexisting autoimmune diseases, history of neck irradiation, prior thyroid surgery or radioactive iodine ablation, drug therapies such as lithium and amiodarone, dietary factors (iodine excess or deficiency) and certain chromosomal or genetic disorders (Turner syndrome, Down syndrome and mitochondrial disease).^5,19^ If initial TSH testing is normal, repeat testing is usually unnecessary unless there is a change in clinical condition.^5,19^

#### Subclinical hypothyroidism

Subclinical hypothyroidism refers to the clinical situation where plasma TSH is elevated despite a normal plasma free T4.^3^ Typically, this is associated with nonspecific clinical symptoms, or no symptoms whatsoever.^3,20^ While elevated TSH levels (particularly > 10 mU/L) are associated with increased cardiovascular and mortality risks, there is insufficient evidence to show that treatment meaningfully affects clinical endpoints.^19,21,22,23,24,25,26,27,28^ Furthermore, the TSH of many patients who initially display biochemical subclinical hypothyroidism will normalise without intervention.^29,30,31^

Of asymptomatic people with an elevated TSH, 62% had normal TSH levels at the second determination, versus only 2.9% who had a second highly elevated TSH.^31^ In addition, thyroid overtreatment can be associated with harms including cognitive dysfunction, atrial fibrillation and bone loss in postmenopausal women.^3,32^ The treatment of asymptomatic adults for screen-detected hypothyroidism is controversial,^19^ and according to the SEMDSA and ACE-SA guideline, treatment of patients with cardiovascular disease (CVD), increased risk for CVD, presence of thyroid peroxidase antibodies (TPO Abs), psychiatric illness, type 2 diabetes, dyslipidaemia or symptoms can be considered.^19^ The British Medical Journal Rapid Recommendations also discourage thyroid hormone therapy for patients with subclinical hypothyroidism.^33^ Treatment for subclinical hypothyroidism is generally recommended when TSH rises above 10 mU/L in patients less than 70 years of age.^1,5^ This is based on a subgroup analysis of a meta-analysis which suggested lower all-cause and cardiovascular mortality with thyroid hormone therapy in that age group.^25^

#### Hypothyroidism in pregnancy

If a woman has risk factors for developing thyroid dysfunction, TSH should be tested early in pregnancy.^7^ There is clear evidence of benefit for treating a pregnant woman known to be hypothyroid.^7,21^ Treatment reduces adverse pregnancy outcomes including preterm delivery or miscarriage, and neuropsychological impairment of the offspring associated with hypothyroidism.^34,35^ However, evidence is insufficient to support universal screening for thyroid disease during pregnancy.^36,37,38^ Early studies suggested that subclinical hypothyroidism during pregnancy might increase adverse pregnancy outcomes, including preterm delivery or miscarriage, and neuropsychological impairment of a child.^35,39^ However, treatment for subclinical hypothyroidism failed to show clinical benefit in randomised placebo-controlled trials.^37^ A meta-analysis of three randomised trials in women with subclinical hypothyroidism diagnosed in pregnancy also found no evidence for benefits of levothyroxine therapy on obstetrical, neonatal, childhood intellectual quotient or neurodevelopmental outcomes.^40^ A recent retrospective Canadian cohort study showed that the current pattern of thyroid testing in pregnant women can contribute to overdiagnosis of hypothyroidism and over-treatment during pregnancy and postpartum.^41^ In addition, over-treatment of pregnant women can increase harms such as preterm labour.^42,43^ Hence, in pregnancy, we lack evidence both for universal screening for thyroid disease, and for treatment of subclinical hypothyroidism and both decisions require considered clinical judgement in the context of the patient and their risk factors.

## Recommendations

- Do not perform routine thyroid function screening in asymptomatic adults.
- Use TSH as the initial test for suspected primary thyroid dysfunction and as the test to monitor levothyroxine monotherapy for primary hypothyroidism.
- Wait six weeks before re-checking TSH after therapy adjustments; once stable, annual testing is sufficient.
- Avoid treating asymptomatic screen-detected subclinical hypothyroidism, except when TSH > 10 mU/L in patients under 70 years of age.
- In pregnancy, test TSH only in high-risk women; treat overt hypothyroidism, but avoid routine treatment of subclinical cases.

## Vignette resolution

*You share the patient’s concern about hypothyroidism. Finding nothing to suggest hypothalamic or pituitary dysfunction, you reassure her that the most useful test is a TSH, which you order, checking ‘suspected hypothyroidism’ on the requisition. The TSH result is high, and the lab automatically reports a low free T4. You diagnose primary hypothyroidism, explain why testing for anti-TPO antibodies is not useful, and start levothyroxine, with a plan to re-check TSH and clinical status in 6–12 weeks.*

## Summary and conclusions

- Routine screening with thyroid function tests is unnecessary in asymptomatic adult patients.
- If thyroid dysfunction is suspected, clinicians should generally begin diagnostic testing with a TSH test alone.
- Use TSH alone to monitor therapy for people taking levothyroxine monotherapy for primary hypothyroidism. Once TSH is within the normal range for age, annual re-checks are appropriate in the absence of clinical changes.
